# Digitale Führungskommunikation und organisationale Bindung von Beschäftigten im Homeoffice

**DOI:** 10.1007/s11612-023-00676-7

**Published:** 2023-04-12

**Authors:** Yasemin Ilter, Faye Barth-Farkas, Tobias Ringeisen

**Affiliations:** FB 3 Allgemeine Verwaltung, Hochschule für Wirtschaft und Recht Berlin, Berlin, Deutschland

**Keywords:** Homeoffice, Digitale Kommunikation, Zugehörigkeitsgefühl, Führungskräfte, Mitarbeitende, Home office, Digital communication, Sense of belonging, Leaders, Employees

## Abstract

Der vorliegende Beitrag der Zeitschrift Gruppe. Interaktion. Organisation. (GIO) untersucht, welche Barrieren die Kommunikation zwischen Führungskräften und ihren Mitarbeitenden im Homeoffice behindern und wie die digitale Führungskommunikation gestaltet werden kann, um das Zugehörigkeitsgefühl von Beschäftigten zum Arbeitgeber zu stärken. Es werden Barrieren und sozial-motivationale Auswirkungen einer digitalen Führungskommunikation im Homeoffice identifiziert und passende Gestaltungsansätze abgeleitet. Verringerte Kontaktmöglichkeiten im Arbeitsalltag, eine erschwerte Emotionswahrnehmung und eingeschränktes Feedback steigern bei digitaler Kommunikation das Risiko einer geschwächten Mitarbeiterbindung, die wiederum mit erhöhter Unzufriedenheit, sinkender Motivation und verringerter Arbeitsleistung der Mitarbeitenden zusammenhängt. Mit Hilfe der Media-Richness-Theorie werden mögliche Kommunikationstools im Überblick vorgestellt, um herauszuarbeiten, welche Medien für welchen Kommunikationszweck im Führungsalltag Anwendung finden können, um die beschriebenen Herausforderungen der digitalen Führungskommunikation zu überwinden. Die Übermittlung von nonverbalen Kommunikationssignalen über Videotools ermöglicht es, Emotionen besser zu transportieren, Nähe einfacher herzustellen und Bindung leichter aufrechtzuerhalten. Eine angemessene Medienwahl hilft der Führungskraft somit, regelmäßigen Kontakt zu Mitarbeitenden zu halten, besser bindungsförderliches Feedback zu geben und eine genauere Wahrnehmung bindungsrelevanter Emotionen zu gewährleisten. Weiterhin ist sinnvoll, die Selbstführung und das Verantwortungsbewusstsein der Beschäftigten im Homeoffice zu stärken. Eine Realisierung der vorgestellten Ansätze sollte durch eine Aufklärung der Mitarbeitenden zu veränderten Prozessen und Rollen im Rahmen einer digitalisierten Führungskommunikation begleitet werden. Zusammenfassend lässt sich eine beziehungsorientierte Führung durch den Einsatz passender Medien auch bei Homeofficetätigkeit der Beschäftigten so gestalten, dass die Mitarbeiterbindung aufrechterhalten und Negativeffekten wie Isolation, sinkender Arbeitsmotivation und verringerter Arbeitsleistung entgegengewirkt wird.

## Abstract (en)

The present article in the journal Group. Interaction. Organization. (GIO) examines which barriers hinder communication between managers and their employees during home office and how digital leadership communication can be adapted to strengthen employees’ sense of belonging. Barriers, challenges and possible solutions for digital management communication during home office are identified. Less contact, difficulties in perceiving emotions and limited feedback while communicating digitally increase the risk of employees feeling less committed to their work and employer. This, in turn, may negatively affect employees’ contentment, motivation, and performance. With the help of Media Richness Theory, potential communication tools are presented in an overview in order to work out which media can be used for which communicative goal in everyday management in order to overcome the described challenges of digital leadership. Communicating through appropriately chosen media allows leaders to remain in regular contact with their employees, give feedback and correctly recognize employees’ emotions, which fosters commitment. Video communication tools are particularly suitable, as the transmission of nonverbal communication signals can better convey emotions, create closeness and maintain attachment to the employer. Furthermore, the article recommends strengthening the employees’ self-leadership skills and thus fostering their sense of responsibility whilst working remotely. The implementation of these approaches should be accompanied by employee education on respective changes in processes and roles. In summary, relationship-oriented leadership behavior may promote employees’ sense of belonging while working from home and prevent negative effects of isolation, poorer motivation and impaired performance through the use of appropriate media.

## Einleitung

Das Führen über digitale Tools wie E‑Mail, Telefon oder Video gewann in den vergangenen Jahren an Bedeutung im Berufsalltag (Nowotny 2019; Robelski et al. 2018). Die coronabedingte Kontaktsperre beschleunigte seit 2020 in vielen Organisationen die Etablierung digitaler Instrumente zur Führung auf Distanz, da die Mitarbeitenden ihre Arbeit vermehrt im Homeoffice erledigten (Kellner et al. 2020). Digitalisierung und zunehmende Homeofficearbeit begünstigen somit die Etablierung neuer Kommunikationsformen und -medien, führen allerdings zu einer Umgestaltung von Einbindung und Arbeitsprozessen, die viele Beschäftigte als Herausforderung erleben (Krämer und Pfizenmayer 2020). Von Führungskräften erfordern diese Veränderungen ausgeprägte Kompetenzen im Umgang mit digitalen Kommunikationstools, um ihre Mitarbeitenden in diesem Transformationsprozess begleiten und ihre Führungsaufgaben weiterhin adäquat erfüllen zu können (Franken 2019).

Trotz einer intensivierten Nutzung digitaler Tools berichtet die Mehrzahl der Führungskräfte über eine erschwerte Kommunikation mit ihren Beschäftigten im Homeoffice (Statista Research Department 2020). Dies zeigt den Bedarf auf, Barrieren für eine gelungene, digitale Führungskommunikation zu identifizieren, Auswirkungen der Homeofficetätigkeit auf die Beschäftigten zu untersuchen und beide Aspekte bei der Ableitung passender Gestaltungsansätze für die Führungskommunikation zu berücksichtigen. Bisher ist wenig erforscht, welche Effekte sich für die Bindung der Beschäftigten zu ihrer Führungskraft aus der Arbeit im eigenen Zuhause ergeben und welche Rolle die Führungskommunikation mit Hilfe digitaler Medien dabei spielt. Erste Langzeituntersuchungen zeigen, dass der Wechsel zur Arbeit im Homeoffice im Zuge der Pandemie zu einer gesteigerten empfundenen Isolation von Angestellten geführt hat (Van Zoonen und Sivunen 2022). Durch eine regelmäßige Kommunikation mit diversen Medien (Van Zoonen und Sivunen 2022) und Unterstützungsangebote durch den Arbeitgeber lässt sich die empfundene Isolation abschwächen (Bentley et al. 2016), was in Folge das Wohlbefinden und die Arbeitsleistung verbessert. Eine Vereinsamung der Beschäftigten im Homeoffice wird zusätzlich durch eingeschränkten Kontakt zu Kolleg*innen verstärkt (Bansmann 2021; Kellner et al. 2020). Fehlende informelle Gespräche im Flur oder in der Kaffeeküche gefährden die kollegiale Beziehungspflege und die Teambildung (Jämsen et al. 2022; Kellner et al. 2020), schränken die Möglichkeit des Netzwerkaufbaus ein und reduzieren das organisationale Zugehörigkeitsgefühl der Mitarbeitenden (Felfe 2008).

Vor diesem Hintergrund untersucht der vorliegende Artikel, welche Barrieren die Kommunikation zwischen Führungskräften und ihren Mitarbeitenden im Homeoffice behindern, welche sozio-motivationalen Auswirkungen sich für die betroffenen Beschäftigten ergeben und wie die digitale Führungskommunikation gestaltet werden kann, um Mitarbeitende besser in betriebliche Abläufe einzubinden und deren Zugehörigkeitsgefühl zum Arbeitgeber zu stärken. Einbezogen wurden aktuelle englisch- und deutschsprachige Studien, von denen einige explizit eine veränderte Führungskommunikation als Folge der Covid-19-Pandemie untersuchen. Ergänzend werden kommunikationstheoretische Ansätze genutzt, um die Befunde modellgestützt einzuordnen (für einen Überblick siehe Walther 2011). Es lassen sich Implikationen ableiten, um eine beziehungsorientierte Führung mit Hilfe passender Medien bei Homeofficetätigkeit der Beschäftigten so zu gestalten, dass die Mitarbeiterbindung aufrechterhalten und Negativeffekten entgegengewirkt wird.

## Digitale Führung und Mitarbeiterbindung

Im Folgenden wird Führung für präsenzbasierte und digitale Arbeitssettings differenziert und ihre Bedeutung für die Mitarbeiterbindung beleuchtet. Ergänzend werden Kommunikationstools vorgestellt, die bei der digitalen Führungskommunikation zum Einsatz kommen können, wenn Mitarbeitende im Homeoffice tätig sind.

### Führungskommunikation und Bindung

Führung bezeichnet einen Kommunikationsprozess, bei dem Angehörige einer Organisation oder Gruppe absichtlich zielbezogen beeinflusst werden, um die Beziehungen dieser Personen und deren Aktivitäten zu leiten, zu strukturieren und zu fördern (Nerdinger 2019 zitiert nach Rosenstiel und Nerdinger 2020; Yukl 2013). Bei der notwendigen Transformation von traditioneller zu digitaler Führung (Torre und Sarti 2020; im englischsprachigen Raum oft als e‑leadership bezeichnet) wird diskutiert, welche Kompetenzen eine Führungskraft neben grundliegender Medienkompetenz entwickeln sollte (Kauffeld et al. 2016). Multidimensionale Kompetenzmodelle können dabei helfen, Führungskompetenzen für eine digitale Zusammenarbeit zu identifizieren. Beispielsweise illustriert das Kompetenzmodell der Great Eight nach Bartram (2005), wie vielschichtig die Verhaltensfacetten erfolgreicher Führung sind und welche davon benötigt werden, um ein disloziertes Team anzuleiten.

Dieser Beitrag räumt der Kommunikationskompetenz einer Führungskraft (als *Interacting and Presenting *im Great Eight-Kompetenzrahmen bezeichnet) im Vergleich zu den restlichen sieben Kompetenzendomänen – *Leading and Deciding, Supporting and Cooperating, Analyzing and Interpreting, Creating and Conceptualizing, Organizing and Executing, Adapting and Coping, Enterprising and Performing* – eine herausragende Bedeutung ein. Steht *Interacting and Presenting *im Vordergrund, kennzeichnet Führung im engeren Sinne somit die interpersonale Kommunikation zwischen unmittelbaren Vorgesetzten und den ihnen unterstellten Mitarbeitenden (Berger 2018; Widuckel 2015), wobei die Beteiligten wechselseitig Informationen über primär arbeitsbezogene Gedanken, Absichten und Emotionen verbal oder nonverbal austauschen (Talley und Temple 2015). Diese Informationen werden vom Sendenden in *Signale* umgewandelt (codiert), über einen Kommunikationskanal oder mehrere *Kommunikationskanäle* übertragen und vom Empfangenden entschlüsselt (decodiert) (Shannon und Weaver 1964). Digitale Führung realisiert interpersonale Kommunikation ausschließlich mit Hilfe von Informations- und Kommunikationstechnologien (Kellner et al. 2020). Auf dem Weg zu einer digitalen Führung (the „way“ toward e‑leadership, Torre und Sarti 2020) stehen Führungskräfte somit vor der Herausforderung, die zur Domäne *Interacting and Presenting *gehörenden Kompetenzdimensionen und -facetten für die Nutzung digitaler Kommunikationsmedien so weiterzuentwickeln, dass die Bindung der Mitarbeitenden zur Führungskraft und zum Unternehmen bei der Arbeit im Homeoffice nicht leidet.

Es lassen sich drei Arten von Kommunikationssignalen unterscheiden (Argyle 2013; Pürer 2015), denen für die Wirkung der Führungskommunikation auf die Mitarbeitenden eine hohe Bedeutung zukommt (Berger 2018). Verbale Kommunikationssignale werden durch Sprechen und Schreiben übermittelt und hauptsächlich über den auditiven Kanal wahrgenommen. Signale wie Tonfall, Tonhöhe, Betonung oder Sprechpausen, die auditiv wahrnehmbar sind und die sprachliche Kommunikation begleiten, werden als paraverbale Signale bezeichnet. Unterstützend wirken nonverbale Signale der Körpersprache, welche mehrheitlich über den visuellen Kanal wahrgenommen werden und eine zentrale Bedeutung für die Beziehung zwischen Führungskraft und Beschäftigten haben, da sie Gefühle, Einstellungen und Persönlichkeitsmerkmale verdeutlichen sowie Status und Rollenverteilung der Kommunikationspartner*innen regeln (Argyle 2013; Röhner und Schütz 2020). Wichtige Körpersignale umfassen Mimik, Dauer und Intensität des Blickkontakts, Gesten in Form von Körperbewegungen, die Körperhaltung sowie räumliche Nähe.

Interviews mit Mitarbeiter*innen zeigen, dass die Bindung und das Zugehörigkeitsgefühl zur Organisation signifikant durch die Kommunikation der Führungskraft beeinflusst werden können (White et al. 2010). Bindung lässt sich als affektives Commitment – neben kalkulatorischem und normativem Commitment – als eine der drei Facetten des organisationalen Commitments konzeptualisieren (Meyer und Allen 1991) und korreliert hoch positiv mit Arbeitszufriedenheit (Klaiber 2018). Felfe (2008) fasst übersichtlich zusammen, wie die Merkmale der Arbeit und der Organisation, sowie individuelle (Persönlichkeits‑)Merkmale der Mitarbeitenden und die Art der Mitarbeiterführung die Mitarbeiterbindung beeinflussen. Die Führungskraft kann eingeschränkt aber signifikant besonders durch einen authentischen Führungsstil (Nasab und Afshari 2019) Einfluss auf das affektive Commitment ihrer Angestellten ausüben.

Auch wenn Führungskräfte die Mitarbeiterbindung nicht ausschließlich durch ihre Kommunikationskompetenz beeinflussen können, konzentriert sich dieser Beitrag besonders auf diesen Faktor, da in der digitalen Zusammenarbeit Kommunikationssignale anders übertragen und kodiert werden als in Face-to-Face Gesprächen. Passen verbale, paraverbale und nonverbale Signale in der Kommunikation der Führungskraft nicht zusammen, so achten Weisungsempfangende in hierarchischen Beziehungen vor allem auf nonverbale und paraverbale Signale; beide Signalarten haben somit einen Einfluss auf die Bindung der Mitarbeitenden zur Führungskraft (für einen Überblick s. Felfe 2008). So können sich wahrgenommene nonverbale und paraverbale Signale der Führungskraft, wie zum Beispiel ein Stirnrunzeln, veränderter Blickkontakt oder eine erhobene Stimmlage, sowohl positiv als auch negativ auf die von Mitarbeitenden erlebte Verbundenheit zur Führungskraft auswirken (Felfe 2008).

Mitarbeiterbindung wird nach der Metastudie von Mathieu und Zajac (1990) durch Merkmale des Arbeitgebers, die Arbeitsbedingungen, Eigenschaften der Führungskraft und individuelle Charakteristika der Angestellten beeinflusst. Mitarbeiterorientiertem Führungsverhalten, welches die Bedürfnisse der Beschäftigten nach Wertschätzung, Zugehörigkeit und Weiterentwicklung befriedigt und mit einer besseren Arbeitsleistung und erhöhter Zufriedenheit assoziiert ist, wird hier eine besonders wichtige Rolle zugeschrieben (Felfe 2008). Organisationsintern umfasst bindungsförderliche Führung das Ausdrücken von Anerkennung, das Anbieten von Unterstützung bei fachlichen und persönlichen Anliegen, die aktive Förderung der beruflichen Weiterentwicklung sowie die Stärkung einer positiven Gruppenidentität (Riggle et al. 2009). Nach außen kann das Bedürfnis nach Bindung durch die Pflege von Netzwerken befriedigt werden, welche den Mitarbeitenden Möglichkeiten zur sozialen Unterstützung bieten und die Lösung berufsbezogener Probleme vereinfachen (Felfe 2008). Vor diesem Hintergrund umfasst bindungsförderliches Verhalten nach Yukl (2012) auch organisationsexternes Kommunikationsverhalten, bei der die Führungskraft für sich und ihre Mitarbeitenden Netzwerke aufbaut und den Arbeitgeber nach außen vertritt. Je differenzierter und fachlich vielfältiger die aufgebauten Netzwerke ausgeprägt sind, desto erfolgreicher gestaltet sich für die Mitarbeitenden die Bewältigung organisationaler Probleme (Felfe 2008).

### Führung von Beschäftigten an digitalen Arbeitsplätzen

In den letzten Jahren haben sich verstärkt virtuelle Arbeitsformen etabliert, bei denen die Mitarbeitenden ihrer Beschäftigung örtlich disloziert nachgehen und der Austausch mit Kolleg*innen, Führungskräften und Externen durch Informations- und Kommunikationstechnik realisiert wird (Frodermann et al. 2020; Kaiser 2020; Kellner et al. 2020). Es lassen sich drei Varianten unterscheiden (ifaa – Institut für angewandte Arbeitswissenschaft e.V. 2019). *Mobile Arbeit* beschreibt, dass Beschäftigte ihrer Tätigkeit örtlich flexibel nachgehen können. *Homeoffice* beschreibt eine eingeschränkt flexible Arbeitsform, bei der teilweise oder ausschließlich zuhause gearbeitet wird (Sandrock et al. 2021). Bei der *Telearbeit *handelt es sich um eine gesetzlich definierte, durch Informations- und Kommunikationstechnik gestützte Arbeitsweise, die außerhalb des betrieblichen Arbeitsplatzes erfolgt. Beschleunigt durch die Covid-19-Pandemie hat in der jüngeren Vergangenheit vor allem das Homeoffice eine hohe Verbreitung erfahren, um die Vorgaben nach sozialer Distanzierung umzusetzen (Hans-Böckler-Stiftung 2021).

Zu den Rahmenbedingungen digitaler Arbeitsplätze gehören *technische Voraussetzungen* im Sinne einer geeigneten Hardwareausstattung mit Computer, Mikrofon und Webcam sowie *strukturelle Voraussetzungen* im Sinne von Regelungen zu Arbeitszeiten und -prozessen (Bruhn 2020; Herrmann und Frey Cordes 2020). Die technische Ausstattung variiert je nach Sektor und Branche: Während beispielsweise eine Umfrage unter Beschäftigten und Führungskräften der Metall- und Elektroindustrie aufzeigt, dass notwendige Hilfsmittel vom Unternehmen gestellt werden (Sandrock et al. 2021), schätzen Beschäftigte der öffentlichen Verwaltung die technische Ausstattung im Homeoffice mehrheitlich als unzureichend ein (Siegel et al. 2020). Regelungen zur Gestaltung und Dokumentation von An- und Abwesenheitszeiten tragen dazu bei, feste Erreichbarkeitsfenster transparent zu machen und sowohl den Austausch intern mit Kolleg*innen und Vorgesetzten als auch extern mit Kund*innen zu ermöglichen (Sandrock et al. 2021). In Anlehnung an eine Befragung von 289 Führungskräften schlussfolgern Akin und Rumpf (2013) von der Managementberatung Hay Group, dass diese strukturellen Voraussetzungen besondere Bedeutung erlangen, wenn Betroffene ortsunabhängig in virtuellen Teams zusammenarbeiten, diese dabei ein gemeinsames Ziel und eine übergeordnete Aufgabe teilen und durch den Einsatz von Informations- und Kommunikationstechnologien miteinander verbunden sind.

**Figure 1:**
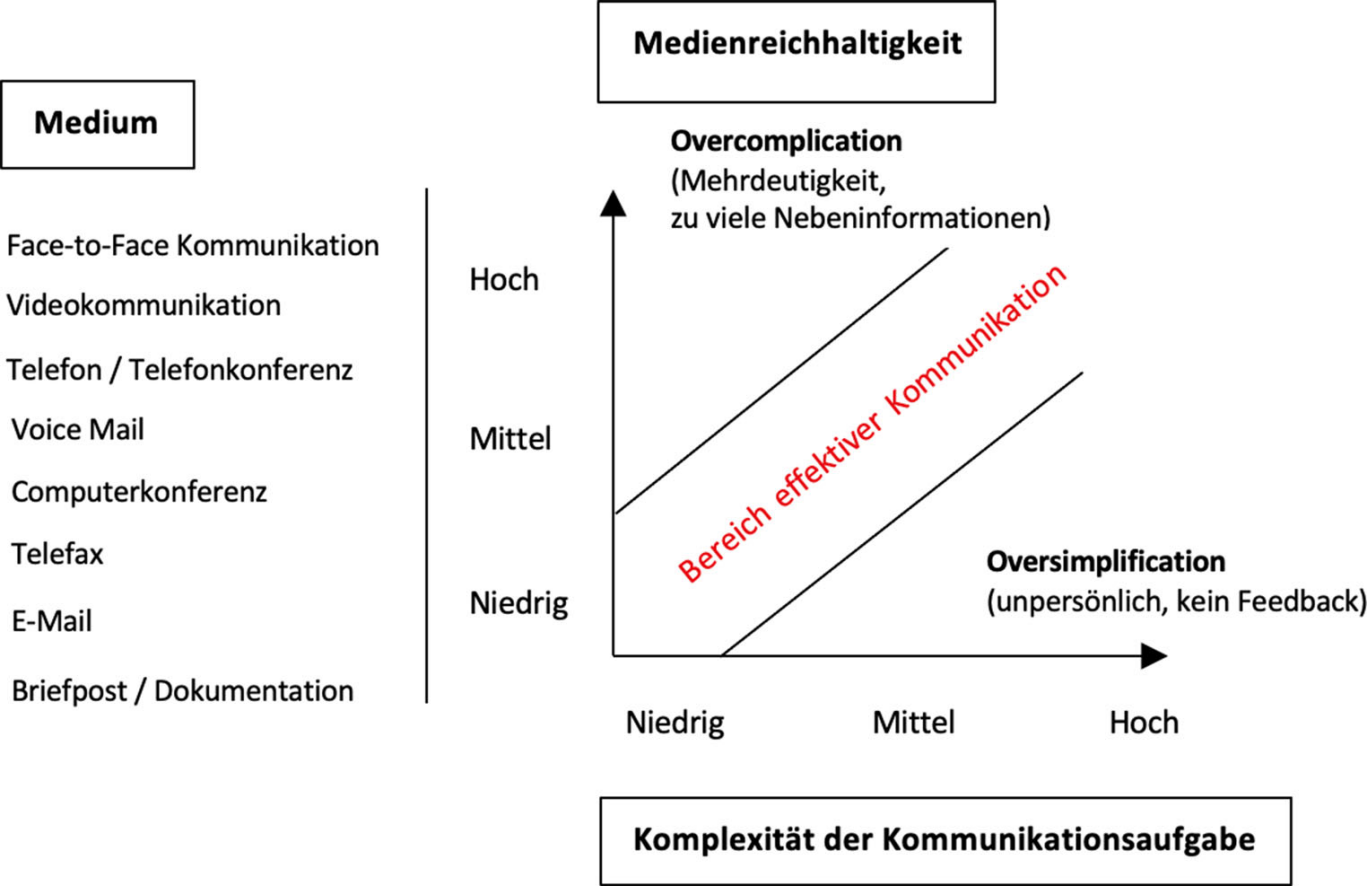

Um die digitale Kommunikation zwischen Vorgesetzten und Beschäftigten zu realisieren, stehen verschiedene Medien zur Verfügung (Bruhn 2020; Kellner et al. 2020), die sich gemäß Media-Richness-Theorie nach ihrer Reichhaltigkeit klassifizieren lassen und passend zu den Anforderungen einer Kommunikationssituation ausgewählt werden sollten (Daft und Lengel 1986). Ist eine Aufgabe durch Kommunikation zu bewältigen, so speist sich deren Anforderung aus ihrer Mehrdeutigkeit bzw. Unsicherheit und ihrer Komplexität. Die Reichhaltigkeit eines Mediums ist umso höher, je (1) schneller eine sofortige Rückmeldung stattfinden kann, (2) je mehr Kommunikationskanäle zur Verfügung stehen, (3) je besser die Vielschichtigkeit von Sprache und Signalen übertragen werden kann und (4) je leichter *sich* persönliche Informationen wie Emotionen oder Einstellungen vermitteln lassen (siehe Abb. 1). Die Media-Synchronicity-Theorie (Dennis et al. 2008) erweitert die Reichhaltigkeit eines Kommunikationsmediums durch den Aspekt der (A)Synchronizität, welcher aufzeigt, ob die Kommunikation der Gesprächspartner*innen zeitgleich oder zeitlich versetzt erfolgt.

Bei Führung im Homeoffice kann ein breites Repertoire digitaler Tools zum Einsatz kommen, deren Reichhaltigkeit systematisch variiert (Bruhn 2020; Klaffke 2019). Für einen bindungsförderlichen Austausch eignen sich Medien wie Videochat am besten, welche in Abhängigkeit der technischen Merkmale eine hohe Reichhaltigkeit erzielen, die an ein Face-to-Face-Gespräch heranreicht. Es stehen eine Vielzahl paralleler Kommunikationskanäle zur Verfügung, die neben der Sprache die bindungsförderlichen para- und nonverbalen Signale wie Betonung, Mimik und Augenkontakt – und somit Emotionen und Einstellungen – gut übertragen (Möslein 1999; Rice 1992). Durch die Möglichkeit einer sofortigen Rückmeldung lassen sich zudem etwaige Missverständnisse reduzieren. Ein einfaches und gängiges Medium ist die Telefonkonferenz, bei der sich zwei oder mehrere Anwesende telefonisch miteinander verbinden. Die Reichhaltigkeit ist als mittel bis hoch einzustufen, da eine sofortige Rückmeldung stattfinden kann, jedoch keine nonverbalen Kommunikationskanäle zur Verfügung stehen. Sowohl Video- als auch Telefonkonferenzen ermöglichen eine synchrone Kommunikation. Bei der Nutzung von Voicemail fehlt die sofortige Feedbackmöglichkeit und der Austausch erfolgt asynchron. Schriftlicher Nachrichtenaustausch kann über E‑Mail, Telefax, kollaborative Arbeitswerkzeuge wie Microsoft Teams oder Online-Formulare erfolgen, die einen effektiven Austausch von Kurznachrichten und Dokumenten im Sinne einer Chat-Funktion ermöglichen. Schriftliche Kommunikation verläuft im Allgemeinen asynchron und weist eine geringe bis mittlere Reichhaltigkeit auf, da Feedback verzögert erfolgt, nur sprachliche Kommunikationskanäle zur Verfügung stehen und para- sowie nonverbale Signale entfallen.

## Herausforderungen der digitalen Kommunikation für eine mitarbeiterorientierte Führung

Führungskräfte sehen sich bei der digitalgestützten Kommunikation mit Beschäftigten trotz reichhaltiger Tools mit vielfältigen Herausforderungen konfrontiert, die im Folgenden näher vorgestellt werden. Unterstützende Faktoren sowie Hindernisse beeinflussen die Effizienz digitalen Führungsverhaltens laut einer Tagebuchstudie mit deutschen Führungskräften während der Pandemie (Krehl und Büttgen 2022). Neben technischen und strukturellen Herausforderungen bei der digitalen Zusammenarbeit empfinden Führungskräfte es vor allem als schwierig, zwischenmenschliche Aspekte wie Teambuilding, Ausdruck von Unterstützung und Feedback sowie Aufbau von Vertrauen zu realisieren (Akin und Rumpf 2013; Krehl und Büttgen 2022). Isolation und nachlassende Bindung werden durch (1) einen verringerten Kontakt zwischen Vorgesetzten und Mitarbeitenden, (2) abgeschwächte gegenseitige Emotionswahrnehmung sowie (3) eingeschränkte Möglichkeiten, Feedback zu geben und zu erhalten, begünstigt.

Die Kausalität der Zusammenhänge zwischen Bindung bzw. organisationalem Commitment und Arbeitszufriedenheit, -motivation und -leistung wird in empirischen Untersuchungen viel diskutiert. Zusammenfassend lassen die Befunde lediglich den Schluss zu, dass eine geringe emotionale Bindung von Mitarbeitenden mit sinkender Motivation, steigender Unzufriedenheit und verringerter Arbeitsleistung der Mitarbeitenden korreliert (Mottaz 1987; Testa 2001). Leitfadengestützte Interviews mit Mitgliedern virtuell zusammenarbeitender Teams zeigen, dass Führungskräfte den bindungsförderlichen Einsatz von Kommunikationsmedien gezielt erlernen und trainieren müssen (Köppel 2009). Die Verhaltensweisen einer mitarbeiterorientierten Führung, die vielen Führungskräften Face-to-Face leichter fallen (Akin und Rumpf 2013), lassen sich nur eingeschränkt auf den virtuellen Führungskontext übertragen (Köppel 2009).

Eine rein digital gestützte Führung birgt allgemein das Risiko, einen weniger häufigen oder weniger intensiven Austausch zwischen Vorgesetzen und Beschäftigten zu begünstigen. Führungskräfte bemängeln, dass dieser *verringerte Kontakt zwischen Vorgesetzten und Mitarbeitenden* dazu beiträgt, dass sich Mitarbeitende im Homeoffice alleingelassen fühlen und ihre Bindung zur Führungskraft sinkt (Bansmann 2021). Zusätzlich bemängeln Berufspraktiker*innen, dass der digitale Austausch zwischen Führungskräften und Mitarbeitenden die Befriedigung der Bedürfnisse nach Anerkennung, Zuneigung und Zugehörigkeit behindert (Aron-Weidlich 2012; Berger 2018; Kaiser 2020). Ergänzend zeigen Tagebuchstudien mit Mitarbeitenden, dass die von ihnen im Homeoffice empfundene Isolation durch wegbrechende informelle Kommunikationswege mit Kolleg*innen verstärkt wird (Kellner et al. 2020).

Die Media-Richness-Theorie legt nahe, dass sich durch den Einsatz der meisten digitalen Kommunikationstools im Homeoffice der Austausch para- und nonverbaler Kommunikationssignale zwischen Führungskräften und Mitarbeitenden reduziert. Folglich werden unter anderem in der Literatur eine erschwerte *Emotionswahrnehmung* und eine verringerte emotionale Unterstützung im digitalen Raum diskutiert (Brosi und Schuth 2020). Eine effektive Emotionswahrnehmung ist dabei multikausal. So empfinden Teammitglieder, die sich bereits gut kennen oder sogar befreundet sind, weniger Schwierigkeiten, die Emotionen der anderen in einer Videokonferenz zu erkennen, als Kolleg*innen, die sich nur wenig kennen (Bleakley et al. 2022). Zusätzlich können sich verschiedene Emotionen der Führungskraft, wie zum Beispiel Wut oder Stolz, unterschiedlich auf die empfundene Distanz der Mitarbeitenden auswirken (Brosi und Schuth 2020). Denkbar ist, dass eine effektive Emotionswahrnehmung daher auch von der entsprechenden Emotion abhängt, die erkannt werden soll. Führungskräfte kritisieren, dass auch bei eingeschalteter Kamera sowohl eigene Nuancen nonverbaler Signale als auch die von Mitarbeitenden verloren gehen (Bansmann 2021). Direkte emotionale Reaktionen und die Körperhaltung sind in Videokonferenzen nicht immer erkennbar, weshalb sich eine Einschätzung der Gefühlslage des Gegenübers häufig auf die wahrgenommene Mimik beschränkt. Als Folge können Führungskräfte schwerer einschätzen, wie sich ihre Beschäftigten fühlen und welche Form der Unterstützung diese benötigen, wodurch sich diese schneller alleingelassen fühlen (Bansmann 2021; Kellner et al. 2020). Besonders ungünstig wirken sich laut einer Onlinebefragung von Angestellten in Deutschland wenig reichhaltige Medien wie E‑Mails aus, die keinen synchronen Austausch zulassen und als unpersönlich wahrgenommen werden (Braun et al. 2019).

Sowohl Führungskräfte als auch Mitarbeitende können im Homeoffice unter verringertem *Feedback* leiden. Kellner et al. (2020) fassen zusammen, dass Mitarbeitende bei ungeregelten Erreichbarkeitszeiträumen im Homeoffice schwerer einschätzen können, wann Kolleg*innen oder Führungskräfte verfügbar sind und dadurch häufiger darauf verzichten, Rückfragen zu stellen und sich stattdessen zurückziehen. Zusätzlich kann es vorkommen, dass Mitarbeitende über textbasierte Kanäle wie E‑Mail und Chat aber auch beim Telefonieren durch den Wegfall von Kommunikationskanälen weniger Feedback von Vorgesetzten wahrnehmen, was die Befriedigung der Bedürfnisse nach Wertschätzung und Weiterentwicklung gefährden kann (Kellner et al. 2020). Zusätzlich können einzelne Merkmale des digital gegebenen Feedbacks durch Führungskräften zu negativen Folgen in virtuell zusammenarbeitenden Teams führen. Eine Metastudie von 2020 zeigt beispielsweise auf, dass eine zeitliche Verzögerung von Feedback oder eine Vernachlässigung von Feedback, welches das ganze Team einbezieht, die meist positiven Auswirkungen von Feedback durch Führungskräfte auf die gemeinsame Zusammenarbeit verringern (Handke et al. 2022). Erschwerend kommt hinzu, dass Mitarbeitende Feedback bei fehlenden para- und nonverbalen Signalen eher als kontrollierendes Verhalten missverstehen (Begerow und Roscher 2020; Yukl 2012). Bei Führungskräften ist fehlendes Feedback der Mitarbeitenden mit einem gesteigerten Misstrauen bei der virtuellen Zusammenarbeit gekoppelt. Beispielsweise begünstigen deaktivierte Kameras bei Online-Meetings und Stummschaltungen bei Telefonkonferenzen das Auftreten von Misstrauen, da Führungskräfte weniger Rückmeldungen von ihren Mitarbeitenden bekommen und deren Aufmerksamkeit im Gespräch nicht überprüfen können (Akin und Rumpf 2013).

## Lösungsansätze für die digitale Führung zur Stärkung der Mitarbeiterbindung

**Figure 2:**
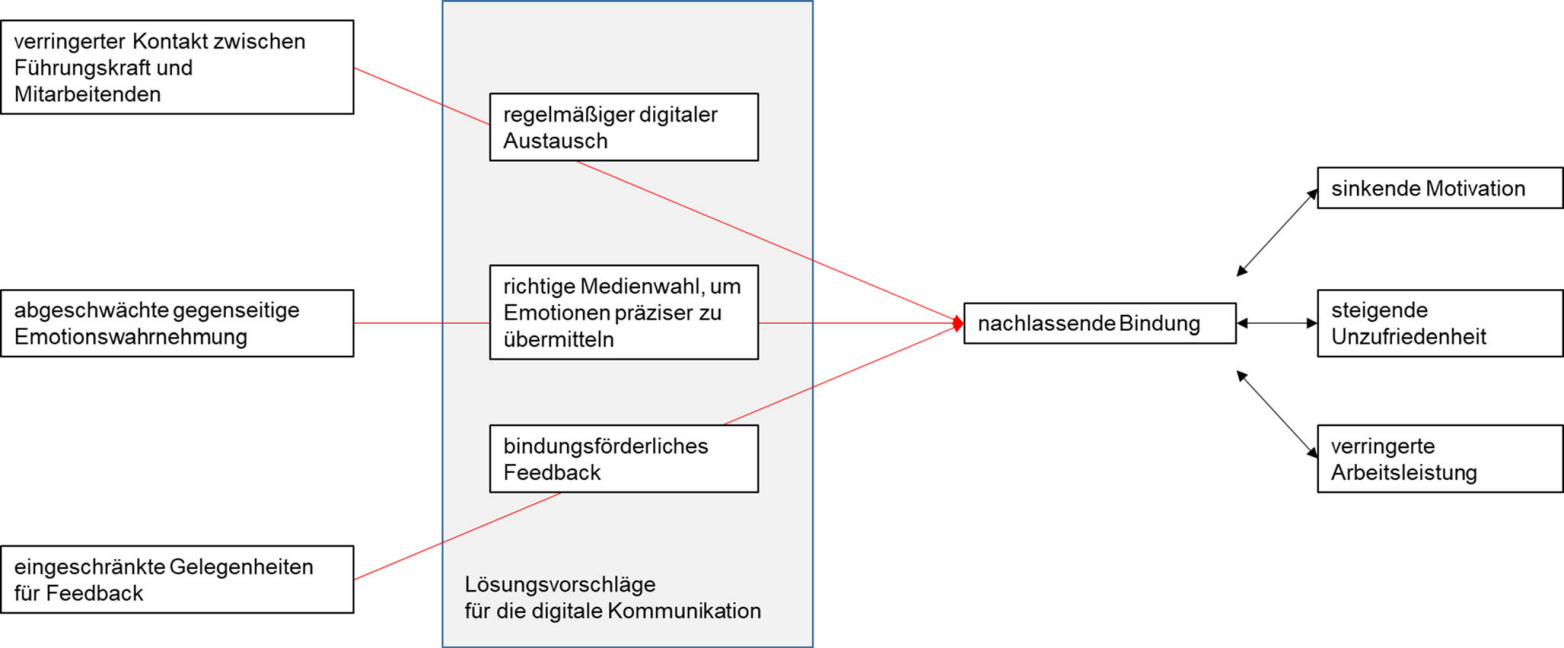

Im Folgenden werden Ansätze für die Führungspraxis vorgestellt, um bei digital gestützter Kommunikation im Homeoffice eine starke Mitarbeiterbindung zu fördern. Neben (1) der Sicherstellung eines regelmäßigen Online-Austauschs zwischen Führungskraft und Beschäftigten können Führungskräfte gestützt auf die MRT Medien mit unterschiedlicher Reichhaltigkeit bewusst aufgabenbezogen auswählen und einsetzen, (2) um gegenseitige Emotionswahrnehmung und das Zugehörigkeitsgefühl ihrer Mitarbeitenden zu stärken und (3) Feedback bindungsförderlich zu gestalten (s. Abb. 2). Damit die bewusst eingesetzte Medienwahl eine hohe Mitarbeiterbindung im Homeoffice begünstigt, sind strukturelle Bedingungen sowie eine gute Vertrauensbasis bei der digitalen Zusammenarbeit unabdingbar (Kellner et al. 2020; Lukić und Vračar 2018).

### Beschäftigten regelmäßig digitalen Austausch mit der Führungskraft ermöglichen

Disloziertes, digitales Arbeiten geht nicht zwangsläufig mit einer reduzierten Kontaktintensität und einem erhöhten Risiko für eine abgeschwächte Bindung von Beschäftigten zu Führungskraft, Kolleg*innen oder dem Arbeitgeber einher. So stellen Wilson et al. (2008) in ihrem Modell der wahrgenommenen Nähe (perceived proximity) fest, dass Mitarbeitende sich durch eine regelmäßige, persönliche und interaktive Kommunikation trotz weiter geografischer Entfernungen sehr verbunden fühlen können. Schon seit Ende der 1990er-Jahre besteht Konsens, dass Führungskräfte durch einen aufgabenbezogenen und flexiblen Einsatz von digitalen Medien die von Beschäftigten erlebte Bindung fördern können (Reichwald und Bastian 1999). Verfügbarkeit, Vertrautheit mit der Nutzung sowie Reichhaltigkeit der gewählten Medien spielen für den Aufbau und Erhalt eines Zusammengehörigkeitsgefühls bei der digitalen Zusammenarbeit eine große Rolle (Wilson et al. 2008). Ergänzend wird Führungskräften empfohlen, ihren Beschäftigten konkrete Erreichbarkeitszeiträume für Anfragen mitzuteilen oder feste virtuelle Sprechstundenzeiten anzubieten, für die sich Interessierte eintragen können. Klare Erreichbarkeiten von Führungskraft und Mitarbeitenden tragen positiv zu einem Teamgefühl bei und mindern den Eindruck, dass jede/r für sich alleine arbeitet (Antoni und Syrek 2017). Idealerweise vereinbaren Führungskräfte mit ihren Beschäftigten im Sinne eines Jour fixes regelmäßige Zeitfenster für einen digitalen Austausch, um die Hemmschwelle für Mitarbeitende zu senken, Kontaktanfragen zu stellen und Störungen zu vermeiden (Aron-Weidlich 2012; Kellner et al. 2020).

### Emotionswahrnehmung und Mitarbeiterorientierung durch die richtige Medienwahl stärken

Geht es um persönliche Anliegen von Beschäftigten, beispielsweise in einer offenen Sprechstunde, oder um eine Reflexion der Zusammenarbeit, beispielsweise in einem Mitarbeitergespräch, so werden Führungskräften reichhaltige Medien wie Videotools zur Stärkung der Mitarbeiterbindung empfohlen (Braun et al. 2019; Lindner und Greff 2019). Videokommunikation erlaubt den synchronen und simultanen Austausch verbaler, nonverbaler und paraverbaler Signale zwischen den Gesprächspartner*innen, wodurch die Emotionslage besser erkennbar ist (Rice 1992). Trotzdem kann es auch im digitalen Videoraum zu einer eingeschränkten Emotionswahrnehmung kommen. Beispielsweise behindern technische Probleme wie ungünstige Beleuchtung, schlechte Bildübertragung oder schwache Bildauflösung das wechselseitige Erkennen von Emotionen. Auch das Arrangement des digitalen Settings kann die Emotionswahrnehmung erschweren, wenn beispielsweise eine Führungskraft ihren Blick auf einen zweiten Bildschirm richtet und von der Kamera abwendet, womit Aufmerksamkeit und Interesse für die Mitarbeitenden weniger deutlich werden (Bleakley et al. 2022).

Treten diese hinderlichen Rahmenbedingungen auf, ist die Führungskraft angehalten, ihre Kommunikationssignale bei Bedarf während der Videokonferenz anzupassen, um besonders dem Vorwurf der Emotionslosigkeit entgegenzuwirken (Bansmann 2021). Eine aufrechte Körperhaltung, deutlicheres Lächeln, intensivierter Blickkontakt oder eine ausgeprägte Gestik seien beispielhaft genannt, um die empfundene Nähe positiv zu beeinflussen (Nowotny 2019; Talley und Temple 2015). Neben bewusst eingesetzter Gestik und Mimik kann die Führungskraft durch die bewusste Modulation der Stimmlage während des Gesprächs per Video Interesse und Aufmerksamkeit ausdrücken: Hohe Töne wirken eher höflich, wohlwollend und fragend, während tiefe Töne ernst, selbstsicher und autoritär wirken können (Nowotny 2019). Diese exemplarischen Möglichkeiten, die videobasierte Kommunikation bindungsfördernd zu gestalten, bieten Führungskräften Anregungen und Ansätze zur Selbstreflektion des eigenen Emotionsausdrucks.

Weniger reichhaltige Medien können von der Führungskraft eingesetzt werden, wenn Emotionserkennung und Nähe eine geringere Relevanz im Gespräch haben. Arbeitsrelevante Fragen lassen sich in der telefonischen Kommunikation schnell und mit sofortigem Feedback klären (Braun et al. 2019). Detaillierte Antworten auf Rückfragen oder komplexe Arbeitsaufträge können von Mitarbeitenden hingegen häufig besser in schriftlicher Form aufgenommen werden. Schriftliche Kommunikationsmedien wie E‑Mails dokumentieren und strukturieren eine hohe Informationsflut und geben Führungskraft sowie Mitarbeitenden mehr Zeit zum Formulieren (Kellner et al. 2020; Trevino et al. 1987). Ist in der schriftlichen Kommunikation eine emotionale Reaktion auf eine mitgeteilte Gefühlslage gewünscht, kann diese mit mithilfe von Emojis ausgedrückt werden. Das Verwenden eines lächelnden Gesichts beispielsweise verstärkt gemäß MRT das Erleben von Nähe und kann Missverständnisse verringern (Bruhn 2020).

Führungskräfte beeinflussen die Mitarbeiterbindung jedoch nicht nur mit Hilfe ihrer eigenen Kommunikation, sondern können auch den informellen Austausch unter Kolleg*innen fördern, um das Zugehörigkeitsgefühl zu stärken. Zu diesem Zweck richten Führungskräfte digitale Räume für den informellen Austausch unter ihren Beschäftigten ein (Bansmann 2021; Kaiser 2020; Krämer und Pfizenmayer 2020). Auch wenn der außerberufliche Austausch über Zoom oder Microsoft Teams einige Einschränkungen aufweist, da parallele sowie spontane Gespräche behindert werden, bewerten die meisten Mitarbeitenden ein solches Angebot positiv und nutzen es aktiv (Bansmann 2021; Krämer und Pfizenmayer 2020). Um den bindungsförderlichen Erfolg informeller Austauschräume zu erhöhen, ist die Führungskraft angehalten, demografische Merkmale der Mitarbeitenden, deren individuelle Kommunikationsmuster und deren Erfahrung mit digitaler Kommunikation zu berücksichtigen. Die genannten Merkmale beeinflussen, wie Mitarbeitende kommunikative Einschränkungen im Homeoffice bewerten und in welchem Ausmaß welche Medien zum informellen Austausch genutzt werden (Wilson 2008). Demnach verwenden jüngere Beschäftigte mit Abitur oder Hochschulabschluss häufiger informelle Chat-Systeme als Ältere mit geringerer Bildung (Kellner et al. 2020). Die individuelle Persönlichkeit, der Arbeitsstil und die Erfahrungen von Mitarbeitenden beeinflussen die Wirkungsweise von unterschiedlichen Kommunikationsmedien, was die Grenzen der MRT illustriert (Handke und Kauffeld 2019). Die Erfahrungen, die Mitarbeitende in der Vergangenheit mit unterschiedlichen Medien gemacht haben, können sich auf die wahrgenommene Reichhaltigkeit eines Mediums auswirken (s. Carlson und Zmud 1999 für eine umfassende Diskussion der Channel-Expansion-Theorie). Daher berücksichtigt eine gut kommunizierende Führungskraft auch diese individuellen Aspekte einzelner Angestellten. Eine Führungskraft, die informelle Kommunikation unter ihren Beschäftigten fördert, stärkt deren organisationales Zugehörigkeitsgefühl, trägt zur Teambildung bei und hilft, Konflikte, die in disloziert arbeitenden Teams häufiger auftreten, präventiv zu verhindern (Felfe 2008; Hinds und Mortensen 2005).

### Bindungsförderlich Feedback geben

Mitarbeitende im Homeoffice erleben oft einen Mangel an Feedback von ihrer digital kommunizierenden Führungskraft, was sich negativ auf die empfundene Wertschätzung, die fachliche Entwicklung und die Bindung auswirken kann (Kellner et al. 2020). Feedback, welches auf Vertrauen und gegenseitigem Zuhören basiert, gewinnt bei der digitalen Kommunikation somit besonders an Bedeutung (Cardon et al. 2019). Verlässliche Erreichbarkeitsfenster einer Führungskraft ermöglichen es den Mitarbeitenden, selbstbewusst Kontakt aufzunehmen und bei Bedarf notwendiges Feedback zeitnah anzufordern (Kellner et al. 2020). Persönliches Feedback sollte über medienreiche Kanäle wie Videokonferenzen erfolgen. Hier können nonverbale Signale besser erkannt werden, wodurch sich Missverständnisse minimieren lassen und Feedback als Entwicklungsinstrument statt als kontrollierendes Verhalten aufgefasst wird (Begerow und Roscher 2020; Yukl 2012). Auch über moderne Chat-Systeme wie das Instant Messaging kann eine positive Feedbackkultur gefördert werden (Kahai et al. 2012). Demnach inspirieren Führungskräfte, die aktiv und regelmäßig kommunizieren, zusätzlich wertschätzendes Feedback unter Kolleg*innen; sie arbeiten zudem häufig in Organisationen mit einer starken Mitarbeiterbindung (Cardon et al. 2019). Ein bindungsförderliches Feedback kann zusätzlich dadurch unterstützt werden, dass Mitarbeitende fortlaufend Einblick in ihre persönliche Leistung und den Forstschritt von Projekten bekommen. Das Potenzial eines persönlichen Feedbacks von der Führungskraft wird voll ausgeschöpft, wenn Mitarbeitende sich parallel selbstständig durch Online-Tools einen Überblick über ihre eigene Leistung verschaffen können (Antoni und Syrek 2017).

## Selbstführung der Beschäftigten als Unterstützungspotenzial

Die Realisierung der im vorherigen Kapitel beschriebenen bindungsförderlichen Ansätze ist aus Sicht der Praxis besonders vielversprechend, wenn parallel die Beschäftigten befähigt werden, ihre individuellen Aufgaben im Sinne einer Selbstführung eigenverantwortlich auszuführen und zu steuern (Bruhn 2020). Traditionell aufgabenorientiertes Führungsverhalten wie Planung, Problemlösung, Kontrolle oder eine klare Aufgabenverteilung hingegen spielen für die Mitarbeiterbindung laut einer umfassenden Metastudie kaum eine Rolle (Yukl 2012). Unter Selbstführung versteht man drei Aspekte des selbstgesteuerten Arbeitens: neben der eigenverantwortlichen Zeitplanung delegierter Aufgaben besitzt die selbstführende Person Freiheiten in der Ausgestaltung ihrer Aufgaben sowie in der Definition von Führungszielen (Müller 2005). Virtuelle Teams profitieren von definierten Rollenverteilungen, klar kommunizierten Hierarchien und Leitlinien für komplexere Prozesse. Diese strukturellen Bedingungen helfen der Führungskraft, ausgewählte Führungsfunktionen unter den Mitarbeitenden aufzuteilen (Bansmann 2021). Festgelegte Rollen- und Zielverteilungen schaffen Transparenz und fördern die Bindung der Mitarbeitenden (Bruhn 2020; Landes et al. 2020; Lindner und Greff 2019).

Für eine erfolgreiche Selbstführung ist sowohl ein beziehungsorientiertes als auch ein aufgabenorientiertes Führungsverhalten von Bedeutung. Aus aufgabenorientierter Perspektive können sich Führungskräfte mit ihren Mitarbeitenden beraten, um deren Vorschläge und Ideen in Entscheidungsprozessen zu berücksichtigen und geeignete Aufgabenbereiche für eine Delegation zu identifizieren (Yukl 2012). Ein intensiver Austausch zwischen Mitarbeitenden und Führungskraft über deren Bedürfnisse nach Autonomie und Kompetenzerleben ermöglicht zusätzlich, den Beschäftigten Aufgaben nach ihren Fähigkeiten zu übertragen und deren Wünsche nach Selbstständigkeit oder Anleitung dabei zu respektieren (Gatt und Jiang 2021; Landes et al. 2020). Wird Selbstführung fähigkeits- und bedürfnisbezogen umgesetzt, trägt diese nicht nur zu einer höheren Entscheidungsqualität, sondern auch zu einer Steigerung der Arbeitszufriedenheit, der Akzeptanz sowie des Commitments unter den Beschäftigten bei (Klaiber 2018). Im Sinne einer mitarbeiterorientieren Führung steigert eine bewusst umgesetzte Selbstführung zusätzlich die empfundene Wertschätzung auf Seite der Mitarbeitenden und kann somit die Herausforderungen durch verringerten Kontakt, eingeschränkte Emotionswahrnehmung und Feedback mildern.

Trotz dieser Positiveffekte wird Selbstführung im Homeoffice bisher zu selten realisiert (Kellner et al. 2020). Viele Arbeitgeber vergrößern den Handlungsspielraum ihrer Beschäftigten nur zögerlich, obwohl sich diese flexible und eigenständig festlegbare Arbeitszeiten wünschen (Zern-Breuer et al. 2020). Führungskräfte haben die Option, Entscheidungen über Verfügbarkeiten und Erreichbarkeiten eigenverantwortlich an Mitarbeitende zu übergeben. Nutzen Mitarbeitende und Führungskräfte beispielsweise im Homeoffice Chat-Systeme oder einsehbare Kalender, können Missverständnisse minimiert und wechselseitiges Vertrauen ausgedrückt werden. Die empfundene Wertschätzung der Mitarbeitenden sowie das Commitment zur Führungskraft wird unter diesen Arbeitsbedingungen gestärkt (Kellner et al. 2020; Landes et al. 2020). Zusätzlich steigt unter diesen Gegebenheiten die Wahrscheinlichkeit, dass Führungskräfte bereit sind, Verantwortung abzugeben und Handlungsspielräume der Beschäftigten auszuweiten.

Eine Selbstführung im Homeoffice, die auch tatsächlich als solche wahrgenommen wird, setzt somit gegenseitiges Vertrauen zwischen Führungskraft und Mitarbeitenden voraus (Nowotny 2019). Da sich der positive Einfluss einer regelmäßigen und offenen Kommunikation auf das Vertrauen in virtuell zusammenarbeiteten Teams im Vergleich zu Präsenzteams abschwächt (Lukić und Vračar 2018), kommen der geeigneten Medienwahl und einer individuellen Betrachtung der jeweiligen Bedürfnis- und Fähigkeitskonstellationen große Bedeutung zu. Um den Erfolg der Selbstführung im Team zu fördern, sollten individuelle Ziele mit den Teamzielen verknüpft werden (Antoni und Syrek 2017). Zusätzlich ist es notwendig, dass die Führungskraft frühzeitig gemeinsam mit den Mitarbeitenden klärt, welches Teamziel mit welchem Zeitplan welche Aufgaben adressiert, und mit Hilfe welcher Medien regelmäßig über den Umsetzungsstand kommuniziert wird (Akin und Rumpf 2013; Landes et al. 2020; Lindner und Greff 2019).

## Fazit und Ausblick

Der vorliegende Beitrag verdeutlicht die erschwerenden Bedingungen, die die emotionale Bindung zwischen Führungskräften und Mitarbeitenden bei digital gestützter Kommunikation im Homeoffice gefährden. Drei zentrale Herausforderungen der digitalen Führungskommunikation lassen sich abgrenzen: der geringere Kontakt zwischen Führungskraft und Mitarbeitenden, die erschwerten Bedingungen für die wechselseitige Emotionswahrnehmung sowie die eingeschränkten Möglichkeiten, Feedback zu geben und zu empfangen. Ohne eine angepasste Führungskommunikation kann aus diesen drei Herausforderungen eine abgeschwächte Mitarbeiterbindung resultieren, welche mit sinkender Mitarbeitermotivation, Zufriedenheit und Arbeitsleistung in Verbindung steht.

Durch eine mitarbeiterorientierte Führung lassen sich potenzielle negative Folgen der drei genannten Herausforderungen reduzieren. Durch regelmäßigen Kontakt, der trotz disloziertem Arbeiten verlässlich aufrechterhalten wird, bleibt die Führungskraft direkte Ansprechpartnerin für Mitarbeitende. Abgesprochene Arbeitszeiten sowie Klarheit über benutzbare Kommunikationsmedien sind hier genauso wichtig wie technische Voraussetzungen für das Arbeiten im Homeoffice. Schwierigkeiten in der Emotionswahrnehmung zwischen Führungskräften, Mitarbeitenden und Kolleg*innen werden durch eine differenzierte Medienwahl gelöst. Kommunikationsmedien, wie das Gespräch per Video, welche nach MRT als besonders reichhaltig gelten und neben verbalen auch para- und nonverbale Signale übertragen, eignen sich gut zur Emotionserkennung beim Gesprächspartner. Eine verbesserte Emotionswahrnehmung kann auch bei digital gestütztem Feedback bindungsförderlich wirken, wenn die Kommunikationswege transparent definiert sind und die Führungskraft aktives Zuhören in einer Videokonferenz praktiziert. Weiterhin wird die emotionale Bindung der Mitarbeitenden durch das informelle Gespräch untereinander gestärkt, welches durch speziell für diesen Zweck entwickelte Apps ermöglicht werden kann. Dabei sind Nutzungsgewohnheiten der verschiedenen Beschäftigtengruppen zu berücksichtigen, da Vertrautheit mit verschiedenen digitalen Medien in Abhängigkeit von demografischen Merkmalen wie Alter, Geschlecht und Bildungsstand die empfundene Zugehörigkeit und das Gefühl von Vereinsamung im Homeoffice beeinflussen können. Ergänzend wirkt eine klare Aufgaben‑, Rollen- und Zielverteilung dem Informationsmangel weniger medienreichhaltiger Kommunikationsmöglichkeiten im Homeoffice entgegen und bildet neben einem hohen Maß an wechselseitigem Vertrauen zwischen Führungskraft und Mitarbeitenden eine wichtige Voraussetzung für eine erfolgreiche Selbstführung von Mitarbeitenden.

Es wird empfohlen, in zukünftigen Studien die Bedeutung ausgewählter, demographischer Variablen, wie z. B. dem Geschlecht, und personenbezogener Variablen, wie z. B. der technikbezogenen Selbstwirksamkeit, auf die Gestaltung der digitalen, bindungssensiblen Kommunikation zwischen Mitarbeitenden und Führungskräften zu untersuchen, da diese Merkmale aktuellen Studien zufolge Emotionen in Online-Settings deutlich beeinflussen können (z. B. Raccanello et al. 2022).

Neben einer Optimierung organisationsinterner Bedingungen für eine gelungene digitale Kommunikation besteht unausgeschöpftes Potenzial, um die digitale Kommunikation mit externen Partner*innen bindungsförderlicher zu gestalten (Siegel et al. 2020). Beispielsweise können Führungskräfte durch den Austausch und die Netzwerkbildung mit unterschiedlichen Unternehmen gegenseitige Erfahrungen und Ideen bei der Arbeit im Homeoffice vergleichen und verbessern.

Auch wenn geeignete Medien lösungsorientiert eingesetzt werden, zeigt sich, dass regelmäßige persönliche Treffen vor allem bei großen Distanzen helfen, Nachteile des virtuellen Raums auszugleichen (Lukić und Vračar 2018). Trotz des großen Spektrums der Kommunikationsmöglichkeiten im Homeoffice zeigen sich hier Grenzen bei der Emotionswahrnehmung. Spontane persönliche Gespräche lassen sich auch durch die Wahl passender digitaler Medien nicht vollständig ersetzen.
